# Targeted Microinjection and Electroporation of Primate Cerebral Organoids for Genetic Modification

**DOI:** 10.3791/65176

**Published:** 2023-03-24

**Authors:** Lidiia Tynianskaia, Nesil Eşiyok, Wieland B. Huttner, Michael Heide

**Affiliations:** 1 German Primate Center, Leibniz Institute for Primate Research; 2 Max Planck Institute of Molecular Cell Biology and Genetics

## Abstract

The cerebral cortex is the outermost brain structure and is responsible for the processing of sensory input and motor output; it is seen as the seat of higher-order cognitive abilities in mammals, in particular, primates. Studying gene functions in primate brains is challenging due to technical and ethical reasons, but the establishment of the brain organoid technology has enabled the study of brain development in traditional primate models (e.g., rhesus macaque and common marmoset), as well as in previously experimentally inaccessible primate species (e.g., great apes), in an ethically justifiable and less technically demanding system. Moreover, human brain organoids allow the advanced investigation of neurodevelopmental and neurological disorders.

As brain organoids recapitulate many processes of brain development, they also represent a powerful tool to identify differences in, and to functionally compare, the genetic determinants underlying the brain development of various species in an evolutionary context. A great advantage of using organoids is the possibility to introduce genetic modifications, which permits the testing of gene functions. However, the introduction of such modifications is laborious and expensive. This paper describes a fast and cost-efficient approach to genetically modify cell populations within the ventricle-like structures of primate cerebral organoids, a subtype of brain organoids. This method combines a modified protocol for the reliable generation of cerebral organoids from human-, chimpanzee-, rhesus macaque-, and common marmoset-derived induced pluripotent stem cells (iPSCs) with a microinjection and electroporation approach. This provides an effective tool for the study of neurodevelopmental and evolutionary processes that can also be applied for disease modeling.

## Introduction

Investigating the (patho)physiological development and evolution of the cerebral cortex is a formidable task that is hampered by the lack of suitable model systems. Previously, such studies were confined to two-dimensional cell culture models (such as primary neural progenitor or neuronal cell cultures) and evolutionarily distant animal models (such as rodents)^1,2^. While these models are useful for addressing certain questions, they are limited in modeling the complexity, cell type composition, cellular architecture, and gene expression patterns of the developing human neocortex in healthy and diseased states. These limitations lead, for example, to the poor translatability of mouse models of human diseases to the human situation, as described for certain cases of microcephaly (e.g., Zhang et al.^3^). Recently, transgenic non-human primates, which are an evolutionarily, functionally, and morphologically closer model of human neocortex development, have come into focus^4, 5, 6, 7, 8^ as they overcome many limitations of cell culture- and rodent-based models. However, the use of non-human primates in research is not only highly expensive and time-consuming but also raises ethical concerns. More recently, the development of brain organoid technology^9, 10^ has emerged as a promising alternative that solves many of the limitations of previous models^11, 12, 13, 14, 15, 16^.

Brain organoids are three-dimensional (3D), multicellular structures that emulate the main features of the cytoarchitecture and cell-type composition of one or multiple brain regions for a defined developmental time window^11, 12, 13, 14, 17^. These 3D structures are generated either from induced pluripotent stem cells (iPSCs) or, if available for the species of interest, from embryonic stem cells (ESCs). In general, two types of brain organoids can be distinguished based on the methodology used: unguided and regionalized (guided) brain organoids^18^. In generating the latter type of organoids, small molecules or factors are provided that guide the differentiation of the pluripotent stem cells to organoids of a particular brain region (e.g., forebrain organoids)^18^. By contrast, in unguided organoids, the differentiation is not guided by the addition of small molecules but rather relies exclusively on the spontaneous differentiation of the iPSCs/ESCs. The resulting brain organoids consist of cell types representing different brain regions (e.g., cerebral organoids)^18^. Brain organoids combine many key features of brain development with relatively cost- and time-efficient generation from any species of interest for which iPSCs or ESCs are available^11, 12, 13, 14^. This makes brain organoids an excellent model for many kinds of neurobiological studies, ranging from evolutionary and developmental questions to disease modeling and drug testing^15, 16^. However, addressing such questions using brain organoids strongly depends on the availability of different methods for genetic modification.

One key aspect of studying neocortex (patho)physiological development and its evolution is the functional analysis of genes and gene variants. This is usually achieved by (ectopic) expression and/or by knock-down (KD) or knock-out (KO) of those genes. Such genetic modifications can be classified into stable and transient genetic modification, as well as into the modifications being temporally and spatially restricted or not restricted. Stable genetic modification is defined by the introduction of a genetic alteration into the host genome that is passed on to all subsequent cell generations. Depending on the time point of genetic modification, it can affect all the cells of an organoid or can be restricted to certain cell populations. Most frequently, stable genetic modification is achieved in brain organoids at the iPSC/ESC level by applying lentiviruses, transposon-like systems, and the CRISPR/Cas9 technology (reviewed by, e.g., Fischer et al.^17^, Kyrousi et al.^19^, and Teriyapirom et al.^20^). This has the advantage that all cells of the brain organoid carry the genetic modification and that it is not temporally or spatially restricted. However, the generation and characterization of these stable iPSC/ESC lines are very time-consuming, often taking several months until the first modified brain organoids can be analyzed (reviewed by e.g., Fischer et al.^17^, Kyrousi et al.^19^, or Teriyapirom et al.^20^).

In contrast, transient genetic modification is defined by the delivery of genetic cargo (e.g., a gene expression plasmid) that does not integrate into the host genome. While this modification can, in principle, be passed on to subsequent cell generations, the delivered genetic cargo will be progressively diluted with each cell division. Therefore, this type of genetic modification is usually temporally and spatially restricted. Transient genetic modification can be carried out in brain organoids by adeno-associated viruses or by electroporation (reviewed by, e.g., Fischer et al.^17^, Kyrousi et al.^19^, and Teriyapirom et al.^20^), with the latter being described in detail in this article. In contrast to stable genetic modification, this approach is very fast and cost-efficient. Indeed, electroporation can be performed within minutes, and, depending on the target cell population(s), electroporated organoids are ready for analysis within days (reviewed by, e.g., Fischer et al.^17^ and Kyrousi et al.^19^). However, gross morphological changes of the brain organoid, such as differences in size, cannot be detected using this method, as this type of genetic modification is temporally and spatially restricted. This restriction can also be an advantage, for example, in the case of studying individual cell populations within the organoid or the effects on brain organoids at specific developmental time points (reviewed by, e.g., Fischer et al.^17^ and Kyrousi et al.^19^).

A classical approach to study gene function during brain development and evolution is *in utero* electroporation. *In utero* electroporation is a well-known and useful technique for the delivery of gene expression constructs into rodent^21, 22, 23^ and ferret^24, 25^ brains. First, a solution containing the expression construct(s) of interest is microinjected through the uterine wall into a certain ventricle of the embryonic brain, depending on the region to be targeted. In the second step, electric pulses are applied to transfect the cells directly lining the targeted ventricle. This approach is not only limited to ectopic expression or the overexpression of genes, as it can also be applied in KD or KO studies by microinjecting short hairpin (shRNA) or CRISPR/Cas9 (in the form of expression plasmids or ribonucleoproteins [RNPs]), respectively^26, 27^. However, the *in utero* electroporation of mouse, rat, and ferret embryos has the same limitations as described above for these animal models.

Ideally, one would like to perform *in utero* electroporation directly in primates. While this is, in principle, technically possible, *in utero* electroporation is not conducted in primates due to ethical concerns, high animal maintenance costs, and small litter sizes. For certain primates, such as great apes (including humans), this is not possible at all. However, these primates have the greatest potential for the study of human (patho)physiological neocortex development and its evolution. One solution to this dilemma is to apply the electroporation technique to primate brain organoids^28^.

This paper presents a protocol for the electroporation of a subtype of primate brain organoids, primate cerebral organoids. This approach allows the fast and cost-efficient genetic modification of cell populations within the ventricle-like structures of the organoids. Specifically, we describe a unified protocol for the generation of primate cerebral organoids from human (*Homo sapiens*), chimpanzee (*Pan troglodytes*), rhesus macaque (*Macaca mulatta*), and common marmoset (*Callithrix jacchus*) iPSCs. Moreover, we describe the microinjection and electroporation technique in detail and provide "go" and "no-go" criteria for performing primate cerebral organoid electroporation. This approach is an effective tool for studying (patho)physiological neocortex development and its evolution in a model especially close to the human situation.

## Protocol

### 1 Culture of primate iPSCs

NOTE: Due to its robustness, the method presented here can be applied to any primate iPSC line. In this article, we describe cerebral organoid production from human (iLonza2.2)^29^, chimpanzee (Sandra A)^30^, rhesus macaque (iRh33.1)^29^, and common marmoset (cj_160419_5)^31^ iPSC lines. The culture conditions are summarized in Table 1. See the Table of Materials for details related to all the materials, reagents, and equipment used in this protocol.

1. For culturing the respective iPSCs, follow the originally described culture conditions. In general, for the successful generation and electroporation of cerebral organoids, use iPSC lines that have not been cultured for more than 90 passages. Additionally, at the start of cerebral organoid generation, ensure that iPSCs exhibit features of pluripotency with no signs of differentiation.

### 2 Generation of cerebral organoids from primate iPSCs

NOTE: The protocol for cerebral organoid generation is based on a modified version^28, 30, 32, 33^ of the original cerebral organoid protocol^10, 34^ with some species-specific modifications (detailed below).

**Figure 1:**
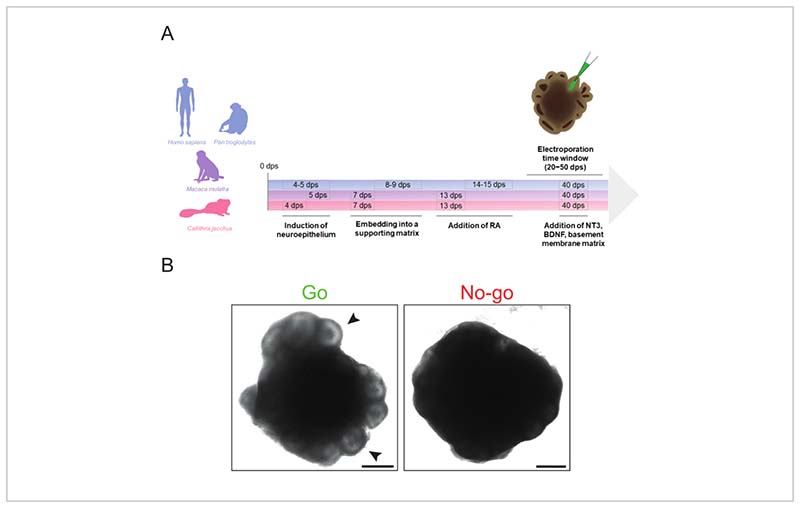
Schematic overview of primate cerebral organoid generation and morphological "go" and "no-go" criteria for electroporation. (**A**) Timeline of primate cerebral organoid generation and electroporation highlighting the different timings of the protocol steps for human and chimpanzee (blue), rhesus macaque (violet), and marmoset (magenta). Note that the chronology of the timeline is not to scale. (**B**) Brightfield images of a suitable (left image, Go) and an unsuitable (right image, No-go) 32 dps human cerebral organoid. The arrowheads indicate examples of suitable ventricle-like structures for microinjection. The images were acquired using a Zeiss Axio Observer.Z1 inverted fluorescence microscope with a 2.5x objective. Scale bars = 500 µm. Abbreviations: BDNF = brain-derived neurotrophic factor; dps = days post seeding; NT3 = neurotrophin 3; RA = retinoic acid.

1. Seeding of iPSCs to generate embryoid bodies (EBs)
  1. Once the iPSCs have reached 80%-90% confluency, wash them with Dulbecco's phosphate-buffered saline (DPBS), and add 500 µL of recombinant trypsin substitute or 1 mL of proteolytic and collagenolytic mixture.
    NOTE: Typically, iPSCs are cultured in a 60 mm cell culture dish to obtain approximately 900,000 cells, which is enough to generate 96 cerebral organoids. The cell numbers can be adjusted depending on the number of organoids needed. Be aware that not all the generated cerebral organoids might be suitable for electroporation.
  2. Incubate the dish at 37 °C for 2 min to detach the cells.
    NOTE: Up to an additional 2 min of incubation at 37 °C might be needed depending on the iPSC line or enzyme used. It is advised to examine the dish under a microscope to ensure that the cells have started detaching.
  3. Add 1.5 mL of prewarmed (37 °C) iPSC culture medium to stop the reaction, and pipette up and down 7x-10x (not more than 10x) to dissociate the cells from the cell culture dish and to obtain a **single-cell suspension**.
  4. Transfer the cell suspension to a 15 mL conical centrifuge tube, and centrifuge the cells at 200 × *g* for 5 min at room temperature.
  5. Aspirate the supernatant, and resuspend the pellet in 2 mL of iPSC culture medium supplemented with either 50 µM Y27632 or 50 µM pro-survival compound.
  6. Use 10 µL of the cell suspension to count the cells using a Neubauer chamber.
  7. Adjust the cell suspension to a concentration of 9,000 cells per 150 µL (60,000 cells/mL) using iPSC culture medium supplemented with 50 µM Y27632 or 50 µM pro-survival compound.
  8. To generate embryoid bodies (EBs), seed 150 µL of the cell suspension into each well of an ultra-low attachment 96-well plate. While pipetting, **gently shake the tube** containing the cell suspension to prevent the cells from sedimenting.
  9. Culture the EBs in a humified atmosphere of 5% CO_2_ and 95% air at 37 °C (**0 days post seeding [dps]**). Do not disturb the EBs within the first 24 h after seeding.
    NOTE: EBs generated from marmoset iPSCs need to be cultured in a humified atmosphere under hypoxic conditions (5% CO_2_, **5% O_2_**, and 90% N_2_) at 37 °C.
  10. After ˜48 h (**2 dps**), change the medium to iPSC culture medium **without Y27632/pro-survival compound**. Remove 100 µL of medium per well, and add 150 µL of prewarmed (37 °C) fresh medium without Y27632. Go row by row.
  11. Perform further medium changes every other day; remove 150 µL of medium from each well, and add 150 µL of prewarmed (37 °C) fresh medium without Y27632/pro-survival compound per well.
    NOTE: After **4-5 dps**, the periphery of the EBs should become translucent.
2. Induction of neuroectoderm
  NOTE: In general, good-quality EBs should have **smooth contours and translucent borders** at this stage. The time points of neural induction slightly differ between primate species and iPSC lines. In the case of the cell lines used here (see section 1 and Table of Materials), neural induction for **marmoset EBs** usually needs to be started **at 4 dps**, for **rhesus macaque at 5 dps**, and for **human and chimpanzee EBs at 4-5 dps** (Figure 1A), depending on the state of the EBs (see above).
  1. Remove 150 µL of medium from each well of the first row of the 96-well plate, and add 150 µL of prewarmed (37 °C) **neural induction medium** (see Table 2) per well in the same row.
  2. Continue changing the medium as described above row by row for the whole 96-well plate. Perform further neural induction medium (NIM) changes every other day by removing 150 µL of NIM from each well and adding 150 µL of prewarmed (37 °C) fresh NIM.
    NOTE: From this point on, the marmoset EBs should be cultured under the same conditions as the other primate EBs (humified atmosphere of 5% CO_2_ and 95% air at 37 °C).
3. Embedding in basement membrane matrix
  NOTE: Once the EBs have developed a pronounced, translucent, radially organized neuroepithelium on the surface, structural support needs to be provided for the development of ventricle-like structures. This is achieved by embedding the EBs into a basement membrane matrix. Due to differences in development rates, **marmoset and rhesus macaque EBs** are ready for embedding already **at 7 dps**, while **human and chimpanzee EBs** are usually embedded at **8-9 dps**. For simplicity, basement membrane matrix refers only to Matrigel in this protocol. However, Geltrex can be used as a replacement.
  1. In preparation for embedding, UV-sterilize scissors, forceps, a small rack for 0.2 mL tubes, and three to six squares of parafilm treated with 70% (vol/vol) ethanol under the laminar flow hood for 15 min. Let the basement membrane matrix thaw on ice for several hours (˜1.5 mL of basement membrane matrix is usually enough for 96 EBs).
    NOTE: **Always** keep the basement membrane matrix on ice.
  2. Create a 4 x 4 dimple grid on the parafilm. Place the parafilm grid on the 0.2 mL tube rack so that the paper-enveloped side is facing up, and gently press a gloved finger against each hole of the rack.
  3. Remove the paper, and cut the dimple grid out of the parafilm square using scissors to adjust its size to fit into a 60 mm cell culture dish. Place the dimpled parafilm back on the 0.2 mL tube rack to provide a basis for the basement membrane matrix droplet generation.
  4. Using a pipette with a cut 200 µL pipette tip, carefully transfer the EBs one after another from the well of the culture dish to the parafilm dimples.
  5. After moving 16 EBs to the grid, take a new 200 µL pipette tip, and remove the remaining medium from the dimples.
  6. Pipette one drop (~15 µL) of basement membrane matrix onto each dimple containing one EB.
  7. Take a 10 µL pipette tip and **quickly** move the EBs into the center of each droplet without disturbing the droplet borders.
  8. Place the dimpled parafilm with the basement membrane matrix drops in a 60 mm cell culture dish, and incubate for 15-30 min at 37 °C to allow the matrix to polymerize.
  9. To detach the matrix-embedded EBs from the parafilm, add 5 mL of **differentiation medium (DM) without vitamin A** (see Table 2) to the dish, and turn the parafilm square upside-down using forceps so that the side with the EBs is facing the bottom of the dish.
  10. Carefully shake the dish to make the basement membrane matrix drops containing the EBs detach from the parafilm. If some of them are still attached, take an edge of the parafilm square using forceps, and rapidly roll it up toward the center of the dish multiple times.
  11. Culture the cerebral organoids on an orbital shaker at 55 rpm in a humified atmosphere of 5% CO_2_ and 95% air at 37 °C. Keep them in **DM without vitamin A** with medium changes every other day. To induce the production of neurons, switch to **DM with vitamin A** (retinoic acid, RA) after **13 dps for marmoset and rhesus macaque cerebral organoids or 14-15 dps for human and chimpanzee cerebral organoids** (Figure 1A). From this point on, change the medium every 3-4 days.
    NOTE: To support neuronal survival, DM with vitamin A can be supplemented with 20 µg/mL human neurotrophin 3 (NT3), 20 µg/mL brain-derived neurotrophic factor (BDNF), and 1 µL/mL basement membrane matrix from 40 dps on.

### 3 Electroporation of primate cerebral organoids

NOTE: From a technical point of view, the electroporation of cerebral organoids can be conducted as soon as the ventricle-like structures are pronounced enough to be targeted by microinjection. The optimal electroporation time window depends on the biological question and on the cell population(s) of interest. For example, if apical progenitors (APs) are the main target, then cerebral organoids at around 30 dps are already suitable. If basal progenitors (BPs) or neurons are the main targets, older cerebral organoids of more than 50 dps should be used (see, for example, Fischer et al.^28^).

1. Preparation of the electroporation setup
  NOTE: The electroporation efficiency is strongly affected by the size and the concentration of the electroporated plasmid(s) (see the discussion section for details).
  1. Prepare a sufficient amount of electroporation mix for the control and gene of interest (GOI), for example, 10 µL of electroporation mix for each control and GOI to electroporate approximately 30 cerebral organoids per condition.
    NOTE: For the composition of the electroporation mixes, see Table 3.
  2. Prewarm (non-sterile) Dulbecco's modified Eagle medium/nutrient mixture F-12 (DMEM/F12) and DM with vitamin A to 37 °C. Prepare a small spatula and fine and normal scissors, and spray the instruments with 70% (vol/vol) ethanol.
    NOTE: The following steps can be performed either under sterile or non-sterile conditions as the DM contains antibiotics (see Table 2). In our experience, the absence of sterility has never caused any contamination.
  3. Prepare 35 mm cell culture dishes, and connect the Petri dish electrode chamber to the electroporator.
    NOTE: Petri dish electrode chambers are commercially available. However, they can be easily produced in a cost-efficient way (see Supplemental File 1).
  4. Using microloader tips, fill microinjection needles with 8 µL of each electroporation mix. Cut the tips of the needles using fine scissors before the first use to achieve a stable flow. However, remove **only a small part of the tip**, as a blunt and wide tip can severely damage the organoids.
    NOTE: Microinjection needles are either commercially available as pre-pulled microinjection needles or can be pulled in the lab if a needle puller is available. Follow the needle puller manufacturer’s instructions to generate microinjection needles with a long taper and a 10 µm tip diameter.
2. Microinjection and electroporation
  1. Under a microscope, choose five cerebral organoids with smooth borders and **clearly visible ventricle-like structures**. Move them to a 35 mm cell culture dish containing prewarmed (37 °C) DMEM/F12 using a cut 1,000 µL pipette tip.
    NOTE: Choose cerebral organoids with pronounced and accessible ventricle-like structures (see Figure 1B).
  2. To inject a ventricle-like structure, carefully insert the needle through its wall, and infuse it with the electroporation mix until **visibly filled**. Do not apply excessive pressure on ventricle-like structures to avoid them bursting. Proceed with six to eight ventricle-like structures of each cerebral organoid in this manner.
    NOTE: If the needle becomes clogged during the microinjection process, the tip needs to be slightly trimmed.
  3. Transfer one microinjected cerebral organoid to the **Petri dish electrode chamber** with a small amount of DMEM/F12. Arrange the organoid in a way that the surfaces of the microinjected ventricle-like structures face toward the electrode connected to the positive pole of the electroporator.
    NOTE: Orienting the structures in this way ensures that the cells are transfected on the side of the ventricle-like structure that is not affected by an adjacent structure.
  4. Electroporate the cerebral organoids one by one using the following settings: **5 pulses** of **80 V**, a **pulse duration** of **50 ms**, and an **interval** of **1 s**. Move the electroporated organoids to a new 35 mm cell culture dish filled with prewarmed (37 °C) DMEM/F12.
    NOTE: The electroporation settings might depend on the available square wave electroporator. These settings are optimized for the referenced electroporation system. Increasing the voltage can lead to the displacement of the cells.
  5. Proceed in the same manner with the next five cerebral organoids using the second electroporation mix (for example, GOI).
    NOTE: Repeat steps 3.2.1-3.2.5 until the desired number of electroporated cerebral organoids is reached.
  6. If the microinjection and electroporation were conducted under non-sterile conditions, transfer the electroporated organoids to a **sterile** 35 mm cell culture dish under a laminar flow hood while moving as little DMEM/F12 as possible to the new cell culture dish.
3. Further culture and fixation of cerebral organoids
  1. Culture the electroporated organoids in DM with vitamin A on an orbital shaker at 55 rpm in a humified atmosphere of 5% CO_2_ and 95% air at 37 °C.
  2. On the next day after electroporation, check the cerebral organoids for successful electroporation under a conventional inverted fluorescence microscope.
    NOTE: Depending on the length of the further culture after electroporation, different cell populations within the cerebral organoid are affected (see also the representative results section).
  3. Proceed with downstream applications after culturing the electroporated cerebral organoids for an amount of time suitable for the biological question of interest.
    NOTE: Electroporated cerebral organoids can be processed for different downstream applications (e.g., fixation for immunofluorescence staining or snap-frozen for RNA isolation and qRT-PCR). Here, we describe the fixation of electroporated cerebral organoids.
  4. Transfer the electroporated organoids to a 15 mL conical centrifuge tube using a cut 1,000 µl pipette tip, and remove excess medium.
  5. Add a sufficient amount of 4% paraformaldehyde (PFA) in DPBS (pH 7.5), and incubate for 30 min at room temperature.
    CAUTION: PFA is classified as a human carcinogen and may cause irreparable health damage. Additional precaution measures including nitrile gloves and goggles are strongly recommended.
  6. Aspirate the PFA, add 5 mL of DPBS, shake a little, and aspirate the DPBS. Repeat this 2x. Store the organoids in DPBS at 4 °C until further use.
    NOTE: The protocol can be paused here, as PFA-fixed cerebral organoids can be stored at 4 °C for several months. PFA-fixed electroporated organoids can be analyzed by cryosectioning and immunofluorescence staining^10, 28^ or by whole-mount staining and clearing^35, 36^. For example images, see the representative results section (see Table 4 for antibody details).

## Representative Results

The protocol described here allows the efficient generation of cerebral organoids from human, chimpanzee, rhesus macaque, and common marmoset iPSC lines with minimal timing alterations required between species (Figure 1A). These organoids can be electroporated in the range of 20 dps to 50 dps, depending on the accessibility of the ventricle-like structures and the abundance of the cell population(s) of interest. However, prior to electroporation, it is important to determine whether the cerebral organoids are of sufficient quality to be electroporated.

A cerebral organoid ideal for electroporation should exhibit pronounced bright ventricle-like structures on the periphery, no signs of degeneration (e.g., detaching cells, enlarged apoptotic core), and a generally compact healthy morphology (e.g., no excessive outgrowth) (Figure 1B, "Go"). It is preferable to choose cerebral organoids with large, well-organized, ventricle-like structures to target a higher number of cells. If the peripheral zone of an organoid is dark and does not show any protruding structures, it is recommended to not use it for electroporation, as the precise microinjections might be compromised by the lack of visual cues (Figure 1B, "No-go"). To achieve an optimal cerebral organoid morphology, it is essential to ensure that critical steps such as the neuroectoderm induction and matrix embedding are well-timed. Problems concerning cerebral organoid morphology typically originate from failed neuroectoderm and/or neuroepithelial bud formation. This is normally caused by suboptimal timing of the neural induction and/or the basement membrane matrix embedding and can be solved by adjusting the timing of these steps (further troubleshooting tips for cerebral organoid formation can be found in Lancaster and Knoblich^34^).

**Figure 2:**
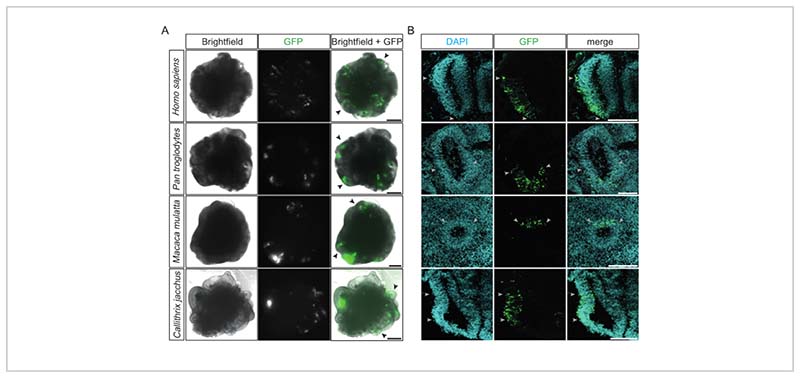
Examples of successfully electroporated primate cerebral organoids. (**A**) Brightfield (left column), fluorescence (middle column), and merge (right column) images of 22 dps human, 32 dps chimpanzee, 32 dps rhesus macaque, and 31 dps marmoset cerebral organoids (from top to bottom) 15-48 h after electroporation with the GFP-expressing plasmid. The black arrowheads indicate examples of individual electroporated ventricle-like structures. The images were acquired using a Zeiss Axio Observer.Z1 inverted fluorescence microscope with a 2.5x objective. Scale bars = 500 µm. (**B**) Immunofluorescence for GFP (green) combined with DAPI staining (cyan) of 32 dps human, 34 dps chimpanzee, 32 dps rhesus macaque, and 32 dps marmoset cerebral organoids (from top to bottom) 2-4 days after electroporation with the GFP-expressing plasmid. The light gray arrowheads indicate the borders of the electroporated regions within the ventricle-like structures. The images were acquired using a Zeiss LSM 800 confocal microscope with a 10x objective. Scale bars = 150 µm. Abbreviations: DAPI = 4',6-diamidino-2-phenylindole; dps = days post seeding; GFP = green fluorescent protein.

After electroporation, a first assessment of its success and its efficiency can be conducted after 12 h, when the GFP expression of the transfected cells becomes detectable under a conventional inverted fluorescence microscope. Ideally, at this stage, multiple ventricle-like structures emit bright green fluorescence localized to one of their sides (Figure 2A). This indicates the high precision and efficiency of the procedure. Successfully electroporated cerebral organoids of the four different primate species (i.e., human, chimpanzee, rhesus macaque, and common marmoset) show similar GFP-positive patterns within the targeted ventricle-like structures (Figure 2A). Moreover, after the fixation and cryosectioning of electroporated primate cerebral organoids, successfully electroporated ventricle-like structures of all four species exhibit columns of GFP-positive cells within the radially organized and densely packed ventricular zone (VZ) (Figure 2B). The quantification of the DAPI-positive cells that were also GFP-positive in such regions of the chimpanzee and marmoset cerebral organoids 2 days post electroporation (17 ventricles from 12 organoids quantified) showed that, on average, roughly one-third of the cells (33%, SD ± 12%) were successfully electroporated.

**Figure 3:**
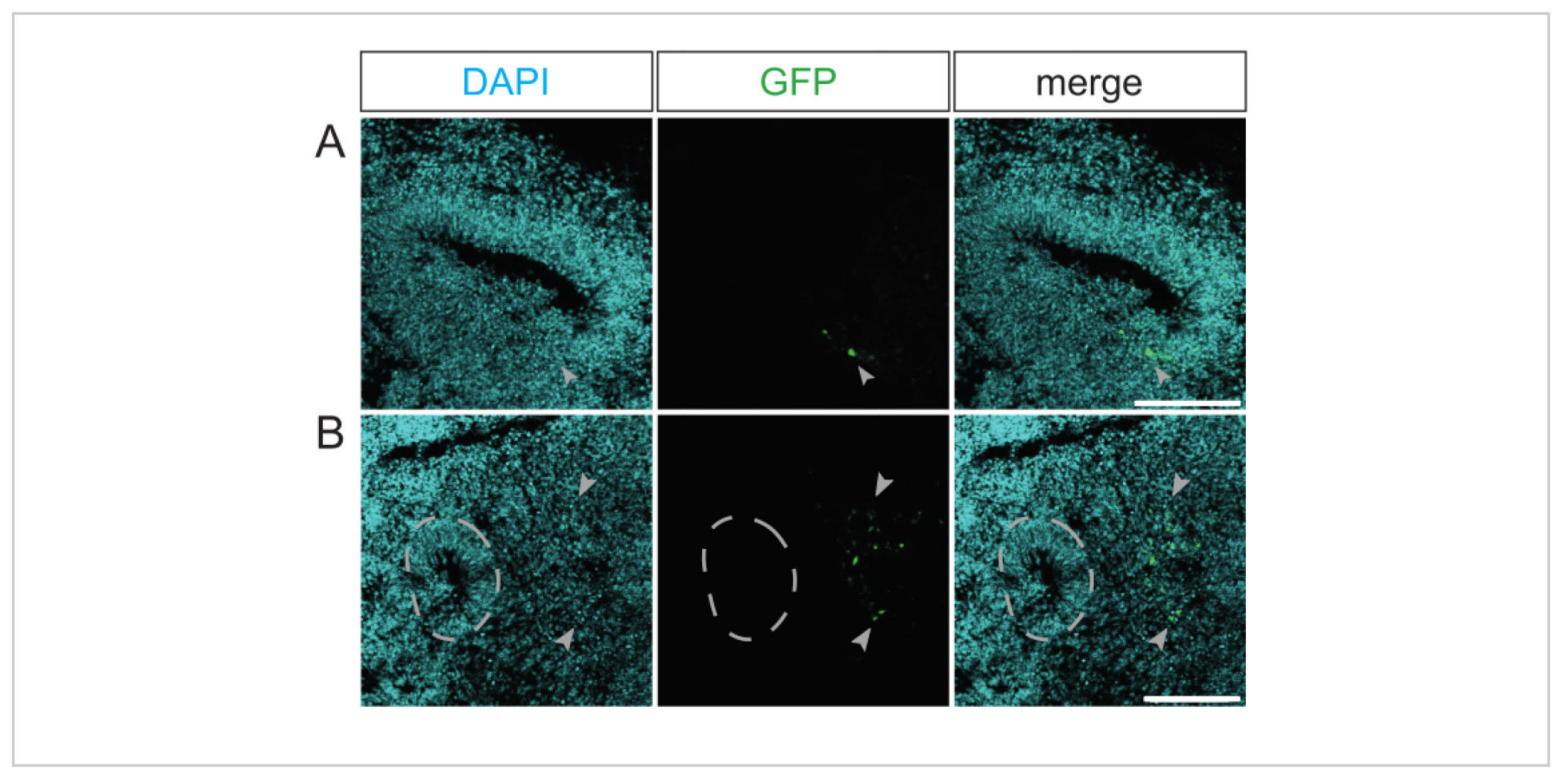
Examples of unsuccessfully electroporated primate cerebral organoids. (**A**,**B**) Immunofluorescence for GFP (green) combined with DAPI staining (cyan) of a (**A**) 34 dps rhesus macaque cerebral organoid 4 days after electroporation with the GFP-expressing plasmid and of (**B**) a 32 dps rhesus macaque cerebral organoid 2 days after electroporation with the GFP-expressing plasmid. The light gray arrowheads indicate electroporated cells. The light gray dashed outline indicates the border between the VZ and SVZ/neuron-enriched zone of a ventricle-like structure adjacent to the electroporated cells. The images were acquired using a Zeiss LSM 800 confocal microscope with a 10x objective. Scale bars = 150 µm. Abbreviations: DAPI = 4',6-diamidino-2-phenylindole; dps = days post seeding; GFP = green fluorescent protein; SVZ = subventricular zone; VZ = ventricular zone.

Suboptimal electroporations are marked either by a small number of GFP-positive cells within the ventricle-like structure (Figure 3A) or by a few GFP-positive cells distant from any ventricle-like structure (Figure 3B). A low number of GFP-positive cells is caused by a poor plasmid uptake. This might be either due to a low plasmid concentration caused by an insufficient amount of microinjected electroporation mix or due to electric pulses that are not well-directed, which may be caused by suboptimal positioning of the cerebral organoids in the Petri dish electrode chamber. A low number of GFP-positive cells distant from any ventricle-like structure is caused by the electroporation of postmitotic cells within the cerebral organoid (e.g., neurons) due to an imprecise microinjection. These suboptimal electroporations need to be excluded from any further analyses.

The reliable identification of the cell types present in cerebral organoids is based, among other things, on the cell position within a ventricle-like structure, which requires a border definition between the VZ and SVZ/neuron-enriched zone. This border can be identified by the radial organization and high cell nuclei density characteristics of the VZ (see DAPI staining in Supplementary Figure S1). The confirmation of the VZ/SVZ border can be performed by immunofluorescence staining for neural progenitor markers such as PAX6 or SOX2, which are expressed by virtually all VZ cells (APs) and some SVZ cells (BPs). The presence of a neuron-enriched zone can be validated by immunofluorescence staining for neuronal markers such as class III β-tubulin (TUJ1) or NeuN (Supplementary Figure S1).

**Figure 4:**
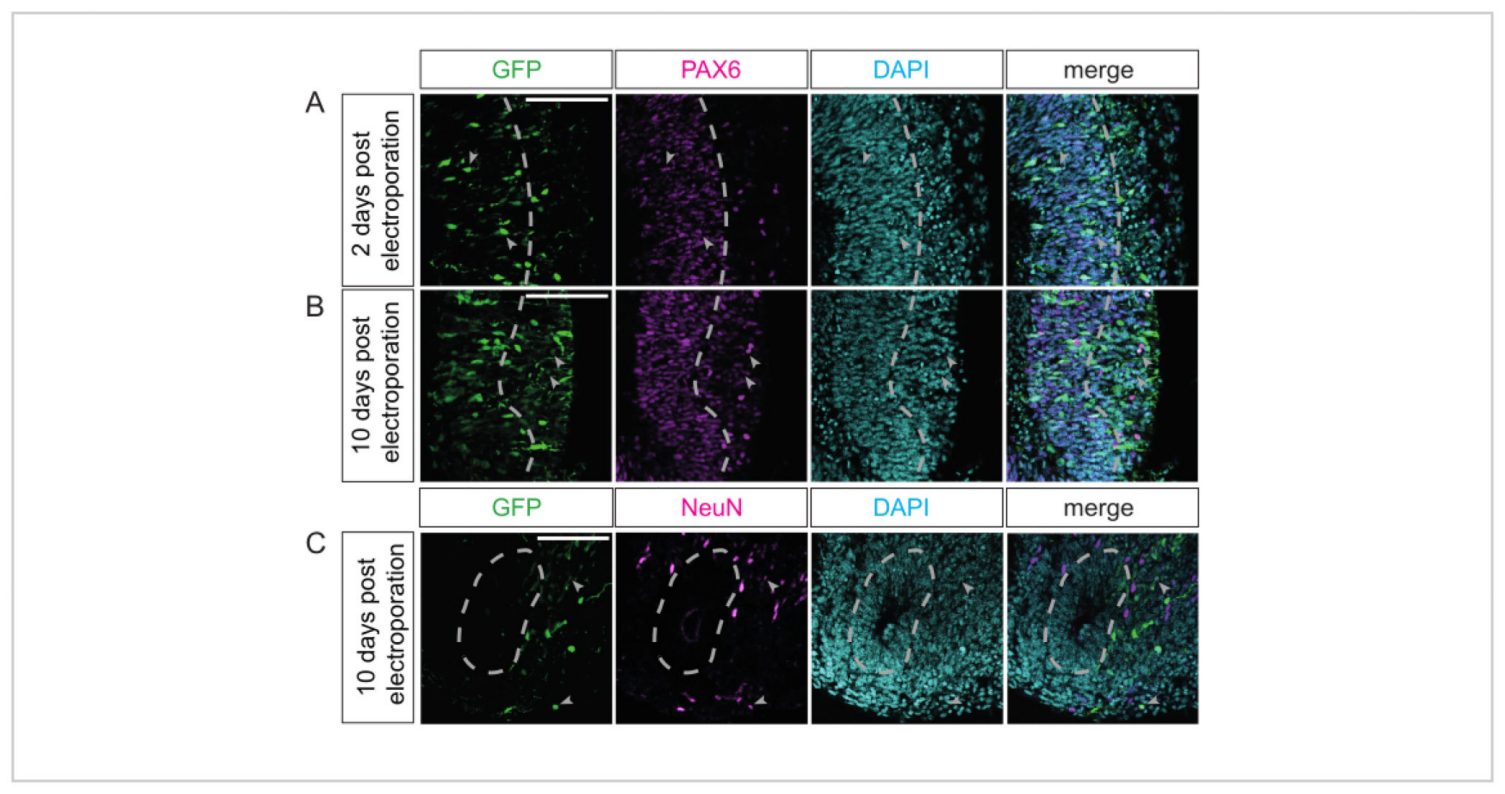
Visualization of the various cell populations present in primate cerebral organoids post electroporation. (**A-C**) Double immunofluorescence for GFP (green) and either PAX6 (**A**,**B**; magenta) or NeuN (**C**; magenta), in all cases combined with DAPI staining (cyan), of a (**A**) 32 dps marmoset cerebral organoid 2 days after electroporation with the GFP-expressing plasmid, and (**B**,**C**) of a 40 dps marmoset cerebral organoid 10 days after electroporation with the GFP-expressing plasmid. The light-gray arrowheads indicate (**A**,**B**) GFP+ and PAX6+ or (**C**) NeuN+ double-positive cells. The light-gray dashed lines indicate the border between the VZ and SVZ/neuron-enriched zone. The images were acquired using a Zeiss LSM 800 confocal microscope with a 20x objective. Scale bars = 100 µm. Abbreviations: DAPI = 4',6-diamidino-2-phenylindole; dps = days post seeding; GFP = green fluorescent protein; NeuN = neuronal nuclei protein; PAX6 = paired box 6 protein; SVZ = subventricular zone; VZ = ventricular zone.

The duration of cerebral organoid culture post electroporation depends on the biological question and the cell populations of interest. In a recent study, it was demonstrated that different lengths of further culture after electroporation affect different cell populations in chimpanzee cerebral organoids, ranging from APs to upper-layer neurons^24^. Here, we show similar results for electroporated marmoset organoids. Specifically, 2 days after electroporation, GFP-positive cells are almost exclusively localized in the VZ and are also positive for PAX6—a marker for neural progenitor cells-indicating that these cells are APs or newborn BPs (Figure 4A). If the culture period after electroporation is extended to 10 days, then GFP-positive cells are localized in the basal regions (i.e., the SVZ and neuron-enriched zone) (Figure 4B,C). These cells can (in addition to the GFP signal) also be positive for PAX6 (Figure 4B), which is indicative of BPs, or NeuN (Figure 4C), which is indicative of neurons. Similar results can be obtained for human and rhesus macaque electroporated cerebral organoids. In summary, different progenitor types, as well as neurons, can be successfully targeted by this technique.

**Figure 5:**
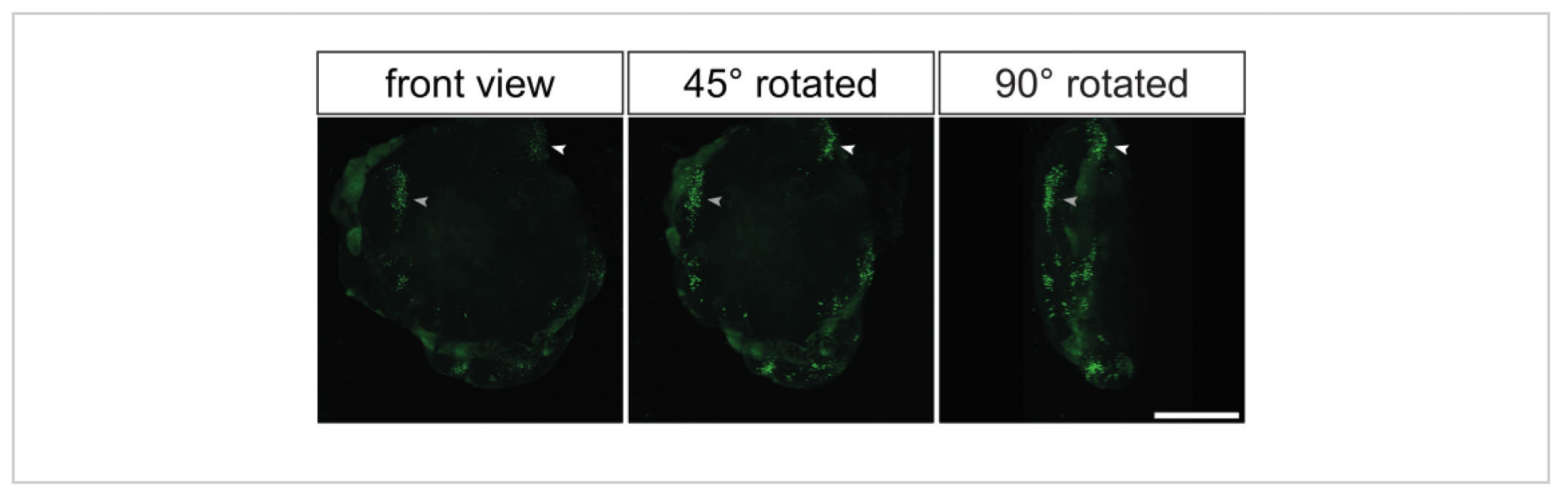
Three-dimensional reconstruction of electroporated images by 3D confocal imaging of electroporated primate cerebral organoids after optical clearing. Frontal (left image), 45° rotated (middle image), and 90° rotated (right image) views of a 3D reconstructed electroporated 32 dps human cerebral organoid 2 days after electroporation with the GFP-expressing plasmid. Prior to imaging, the organoid was optically cleared based on the 2Eci method^35^. A 3D reconstruction of whole electroporated organoid was generated from 269 optical sections (1-µM thickness each) that are 3.73 µm apart from each other using a Zeiss LSM 800 confocal microscope with a 10x objective. The images were processed for 3D reconstruction using Fiji. Note that the images were taken from the same 3D-reconstructed organoid shown in Video 1. Scale bar = 500 µm. Abbreviation: GFP = green fluorescent protein.

Almost all the previously shown data were derived from the immunostaining of histological sections generated from electroporated cerebral organoids. However, another elegant way to analyze these organoids is to perform whole-mount immunostaining followed by optical clearing^35, 36^. This would allow 3D reconstruction of the electroporated cerebral organoids to obtain an impression of the 3D distribution of the GFP-positive cells. Figure 5 and Video 1 show a representative example of the GFP signal in an optical-cleared, electroporated cerebral organoid.

In summary, the electroporation protocol described here provides a precise and efficient way to introduce transient genetic modification(s) into different progenitor types and neurons of cerebral organoids derived from different primate iPSC lines.

## Discussion

The procedures described here represent a unified protocol for the generation of cerebral organoids from different primate species with a targeted electroporation approach. This allows the ectopic expression of a GOI in a model system that emulates primate (including human) (patho)physiological neocortex development. This unified protocol for the generation of primate cerebral organoids uses the same materials (e.g., media) and protocol steps for all four primate species presented. Developmental differences between these species are addressed by changing the timing of critical protocol steps (i.e., the neural induction and embedding in the basement membrane matrix; see above). This might roughly reflect the *in vivo* neurodevelopmental timing differences between these species and constitutes an interesting topic for further studies.

This approach is based on the electroporation experiments described in a previous paper on cerebral organoids^10^. However, these experiments, as noted by Lancaster and colleagues, were limited by extensive degrees of apoptosis, which occurred due to a high GFP concentration and led to the exclusion of the electroporated cells exhibiting a strong GFP signal^10^. In our experiments, we found that a final total plasmid concentration (e.g., EGFP-encoding plasmid concentration plus GOI-encoding plasmid concentration) of **1,000 ng/µL was optimal**. Concentrations lower than 1,000 ng/µL lead to reduced electroporation efficiency, while high concentrations above 1,000 ng/µL could be toxic for the electroporated cells and lead to cell death^10^. Studies combining more than two different expression plasmids are possible^28^. However, the final total plasmid concentration should be kept at 1,000 ng/µL.

In a typical electroporation approach, a plasmid encoding a fluorescence marker is needed to identify the successfully electroporated cells. There are two possibilities for including the fluorescence marker in the experimental setup: (i) by co-injecting two separate plasmids (i.e., a plasmid encoding for the marker plus a control plasmid [e.g., empty vector] or a plasmid encoding the gene of interest [GOI]) (the **separate plasmids approach**); (ii) by injecting one plasmid that encodes for both the marker and the GOI by using an internal ribosome entry site (IRES) or a 2A self-cleaving peptide (e.g., P2A) (the **single plasmid approach**). In this case, a plasmid encoding solely the fluorescence marker is used as a control. While the single plasmid approach results in the full co-expression of the fluorescence marker and GOI, such plasmids are large in size, which results in a low electroporation efficiency. If a high electroporation efficiency is needed, it is **recommended to use the separate plasmids approach**, as the separated expression does not significantly affect the level of co-expression of the fluorescence marker and of the GOI while maintaining a high electroporation efficiency^28^. In the protocol presented here, we describe the electroporation using the separate plasmids approach. If the single plasmid approach is applied, the steps in the protocol must be adjusted accordingly.

In comparison to a recently published protocol^37^, our approach has three main advantages. First, we specifically target the ventricle-like structures of the cerebral organoid. We achieve this (i) by microinjecting the individual ventricle-like structures of the cerebral organoid instead of injecting into the center of the organoid^37^ and (ii) by arranging the orientation of the cerebral organoid in the Petri dish electrode chamber to optimize the direction of the electric field (see above) instead of using an electroporation cuvette^37^. Second, this protocol does not involve the use of an expensive nucleofector solution, as this approach uses a square wave electroporator in combination with a Petri dish electrode chamber. Third, this unified protocol for the generation of primate cerebral organoids allows the study of not only human but also non-human primate organoids, which allows evolutionary, comparative, and disease studies.

Two features concerning the cerebral organoids are critical for a successful electroporation—the quality of the cerebral organoids and the size and the quality of the ventricle-like structures (see the representative results and Figure 1B). Concerning the first feature, we present go and no-go criteria (see above and Figure 1B) for proceeding with the electroporation. The main criterion is the presence of clearly visible, translucent, and radially organized ventricle-like structures. The size of the ventricle-like structures is the second crucial feature for successful microinjection and electroporation. Ventricle-like structures that are too small are difficult to inject and normally do not yield a sufficiently high number of electroporated cells for subsequent analyses. This is the main reason why we used a modified cerebral organoid protocol, as this protocol, in our hands and for these iPSC lines-produces, in comparison to other protocols, well-organized ventricle-like structures that are sufficiently large to be electroporated. In principle, the electroporation approach presented here can be applied to any other neural organoid protocol as long as the latter yields sufficiently large and organized ventricle-like structures (see the representative results and Figure 1B). Moreover, this protocol could also be applied to other primates in the future such as the crab-eating macaque (*Macaca fascicularis*), the classical primate model used in industrial research. This would require either the identification of the correct critical time points (see above) of the modified cerebral organoid protocol described here or the establishment of a neural organoid protocol that gives rise to suitable large ventricle-like structures (see above).

After successful electroporation, the cerebral organoids can be further cultured for different periods of time to allow the genetic modification under examination to affect the various cell populations within the developing cerebral organoid. These range from the various neural progenitor populations to the diverse types of neurons present in the cerebral organoid (see the representative results and Fischer et al.^28^). These cell populations can then be analyzed either by cryosectioning and immunofluorescence staining (see Figure 4) or by whole-mount immunofluorescence staining and optical clearing (see Figure 5 and Video 1) of the PFA-fixed electroporated cerebral organoids.

So far, electroporation of brain organoids has mainly been used for the ectopic expression of genes to perform gene function studies^10, 28, 38, 39^, live imaging^10, 39, 40^, the visualization of cell morphology^40^, and cell division tracing^10^. However, in the first cerebral organoid paper, shRNA was introduced into organoids by electroporation to silence gene expression by RNA interference. This showed the potential of electroporation to be used not only for the ectopic expression of genes but also for the KD or even KO of genes. Recently, it was shown that CRISPR/Cas9-mediated KOs can be achieved by the *in utero* electroporation of mouse embryos^27^ and the electroporation of fetal human tissue *ex vivo*^41^. Such CRISPR/Cas9-based KOs could be easily applied to the electroporation of cerebral organoids, as the principal mechanism of electroporation is the same, and this would further expand the usefulness of this approach.

One potential further application of electroporation in cerebral organoids is the rescue of specific KO phenotypes in cases in which it is not possible to obtain a specific KO of the GOI due to highly similar sequences between the GOI and its ancestral versions. This is particularly the case for recently evolved human-specific genes. In such cases, it is not possible (even with the use of the CRISPR/Cas9-technology) to obtain a specific KO (as was the case for *ARHGAP11A* and *ARHGAP11B*^28^). A solution to this dilemma is to generate a double KO of the GOI and its ancestral gene and to rescue the GOI alone, the ancestral gene alone, or both genes together by electroporation. These electroporated organoids could then be considered as a selective ancestral gene KO, a selective GOI KO, or a control, respectively. This would allow the individual contributions of these genes to the phenotype to be addressed (see, e.g., Fischer et al.^28^). Another potential application is related to the analysis of cerebral organoids generated from patient-derived iPSCs. In such cases, it is not clear if the observed phenotype is due to a mutation in the candidate gene or due to any other mutation present in the patient. Here, the electroporation of the candidate gene and the (potential) rescue of the phenotype would allow the role of this gene in the disease to be validated (see, e.g., Lancaster et al.^10^).

Taken together, the protocol presented here offers a fast and cost-efficient approach to genetically modify cell populations within the ventricle-like structures of primate cerebral organoids. This provides an effective tool for the study of neurodevelopmental and evolutionary processes that can also be applied for disease modeling.

## Supplementary Material

Supplemental Figure S1 VZ/SVZ border determination in electroporated primate cerebral organoids. Double immunofluorescence for PAX6 (magenta) and TUJ1 (yellow) combined with DAPI staining (cyan) of a 32 dps marmoset cerebral organoid 2 days after electroporation with the GFP-expressing plasmid. The immunofluorescence for GFP is not shown. The light-gray dashed lines indicate the border between the VZ and SVZ/neuron-enriched zone. The images were acquired using a Zeiss LSM 800 confocal microscope with a 20x objective. Scale bar = 100 μm. Abbreviations: DAPI = 4',6-diamidino-2-phenylindole; dps = days post seeding; PAX6 = paired box 6 protein; SVZ = subventricular zone; TUJ1 = class III β-tubulin; VZ = ventricular zone.

Video 1 A 3D-reconstructed electroporated human cerebral organoid after optical clearing. Video of a 3D-reconstructed electroporated 32 dps human cerebral organoid 2 days after electroporation with the GFP-expressing plasmid. Prior to imaging, the organoid was optically cleared based on the 2Eci method^35^. A 3D reconstruction of whole electroporated organoid was generated from 269 optical sections (1-μM thickness each) that are 3.73 μm apart from each other using a Zeiss LSM 800 confocal microscope with a 10x objective. The images were processed for 3D reconstruction using Fiji. Note that the video was taken from the same 3D-reconstructed organoid shown in Figure 5.

Table of materials

Supplemental File 1

## Figures and Tables

**Table 1:** Culture conditions for the primate iPSCs used in this publication. Abbreviation: iPSCs = induced pluripotent stem cells.

iPSC line	Species	Publication	Culture medium composition	Culture conditions
iLonza2.2	*Homo sapiens*	Stauske et al., 2020	μM IWR1 and 0.5 μM CHIR in StemMACS iPS-Brew XF	humified atmosphere of 5% CO_2_ and 95% air, 37 °C
SandraA	*Pan troglodytes*	Mora- Bermudez et al., 2016	mTeSR1	humified atmosphere of 5% CO_2_ and 95% air, 37 °C
iRh33.1	*Macaca mulatta*	Stauske et al., 2020	μM IWR1 and 0.5 μM CHIR in StemMACS iPS-Brew XF	humified atmosphere of 5% CO_2_ and 95% air, 37 °C
cj_160419_5	*Callithrix jacchus*	Petkov et al., 2020	3 μM IWR1,0.3 μM CGP77675, 0.3 μM AZD77675, 0.5 μM CHIR99021, 10 μM Forskolin, 1 ng/mL Activin A, 1 μM OAC1 in StemMACS iPS-Brew XF	humified atmosphere of **5% CO_2_, 5% O_2_, and 90% N_2_,** 37 °C

**Table 2:** Composition of the media used for primate cerebral organoid generation and culture.

Medium	Composition
Neural induction medium	1x N-2 supplement, 1x Glutamine substitute Supplement, 1x MEM Non-Essential Amino Acids Solution, 1 μg/mL Heparin in Dulbecco's Modified Eagle Medium F12 (DMEM/F12)
Differentiation medium (DM) without Vitamin A	0.5x B-27 Supplement (minus vitamin A), 0.5x N-2 supplement, 0.5x MEM Non-Essential Amino Acids Solution, 1x Glutamine substitute Supplement, 100 U/mL Penicillin-Streptomycin, 0.00035% 2-Mercaptoethanol, 2.875 ng/mL Insulin in 1:1 DMEM/F12 and Neurobasal Medium
Differentiation medium (DM) with Vitamin A	0.5x B-27 Supplement, 0.5x N-2 supplement, 0.5x MEM Non-Essential Amino Acids Solution, 1x Glutamine substitute Supplement, 100 U/mL Penicillin-Streptomycin, 0.00035% 2-Mercaptoethanol, 2.875 ng/mL Insulin in 1:1 DMEM/F12 and Neurobasal Medium

**Table 3:** Composition of the electroporation mix (separate plasmids approach) for the control and gene of interest. Abbreviation: GOI = gene of interest.

Component	Control electroporation mix	GOI electroporation mix
GFP expression plasmid	500 ng/μL	500 ng/μL
Empty vector	500 ng/μL	-
GOI expression plasmid	-	500 ng/μL
Fast Green	0.10%	0.10%
in DPBS		

**Table 4:** Antibodies used for immunofluorescence staining.

Antibody	Company	Catalog Number	RRID	Dilution
Chicken anti GFP	Aves labs	GFP-1020	RRID:AB_10000240	1:300
Rabbit anti PAX6	Novus Biologicals	NBP1-89100	RRID:AB_11013575	1:300
Rabbit anti NeuN	Abcam	ab104225	RRID:AB_10711153	1:300
Goat anti chicken	Thermo Fisher	A-11039	RRID:AB_142924	1:500
Alexa Fluor 488				
Donkey anti rabbit	Thermo Fisher	A-31572	RRID:AB_162543	1:500
Alexa Fluor 555				
