# Supplementary figures and images for: Targeted Microinjection and Electroporation of Primate Cerebral Organoids for Genetic Modification
Source: J Vis Exp. Author manuscript; Available in PMC 2024 Feb 6. (PMC7615602; doi:10.3791/65176)

2 days post  
electroporation

PAX6

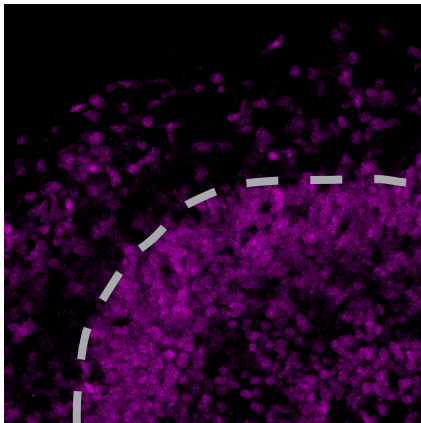

TUJ1

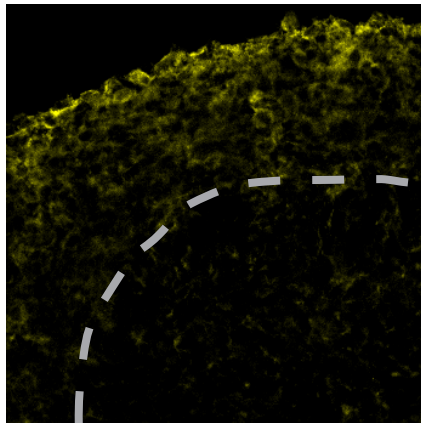

DAPI

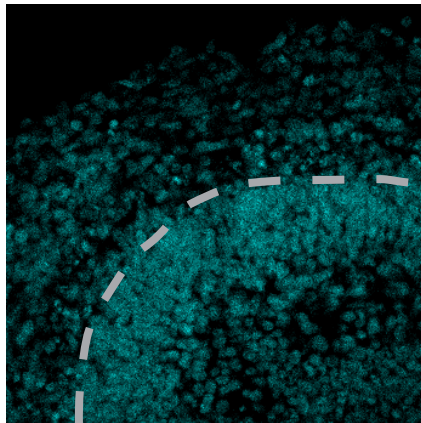

merge

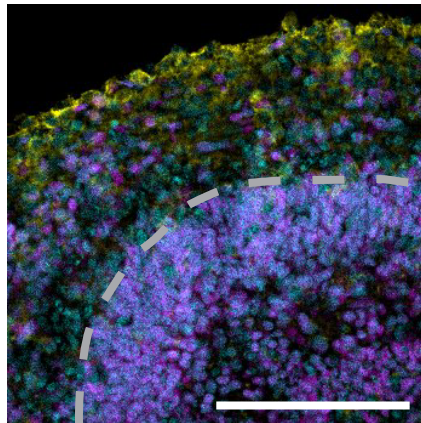

Supplement: Supplemental Figure S1 — Double immunofluorescence for PAX6 (magenta) and TUJ1 (yellow) combined with DAPI staining (cyan) of a 32 dps marmoset cerebral organoid 2 days after electroporation with the GFP-expressing plasmid. The immunofluorescence for GFP is not shown. The light-gray dashed lines indicate the border between the VZ and SVZ/neuron-enriched zone. The images were acquired using a Zeiss LSM 800 confocal microscope with a 20x objective. Scale bar = 100 μm. Abbreviations: DAPI = 4',6-diamidino-2-phenylindole; dps = days post seeding; PAX6 = paired box 6 protein; SVZ = subventricular zone; TUJ1 = class III β-tubulin; VZ = ventricular zone. [file EMS173298-supplement-Supplemental_Figure_S1.pdf]
