# Supplementary material for: Targeted Microinjection and Electroporation of Primate Cerebral Organoids for Genetic Modification
Source: J Vis Exp. Author manuscript; Available in PMC 2024 Feb 6. (PMC7615602; doi:10.3791/65176)
Supplement: Supplemental File 1 [file EMS173298-supplement-Supplemental_File_1.pdf]

Supplemental Figure S1: VZ/SVZ border determination in electroporated primate cerebral organoids. Double immunofluorescence for PAX6 (magenta) and TUJ1 (yellow) combined with DAPI staining (cyan) of a 32 dps marmoset cerebral organoid 2 days after electroporation with the GFP-expressing plasmid. The immunofluorescence for GFP is not shown. The light-gray dashed lines indicate the border between the VZ and SVZ/neuron-enriched zone. The images were acquired using a Zeiss LSM 800 confocal microscope with a 20x objective. Scale bar = 100 µm. Abbreviations: DAPI = 4',6-diamidino-2-phenylindole; dps = days post seeding; PAX6 = paired box 6 protein; SVZ = subventricular zone; TUJ1 = class III β-tubulin; VZ = ventricular zone. Please click here to download this File.

Supplemental File 1: Petri dish electroporation chamber assembly instructions. Please click here to download this File.

Copyright © 2023 JoVE Journal of Visualized Experiments
